# Sanitation and Hygiene-Specific Risk Factors for Moderate-to-Severe Diarrhea in Young Children in the Global Enteric Multicenter Study, 2007–2011: Case-Control Study

**DOI:** 10.1371/journal.pmed.1002010

**Published:** 2016-05-03

**Authors:** Kelly K. Baker, Ciara E. O’Reilly, Myron M. Levine, Karen L. Kotloff, James P. Nataro, Tracy L. Ayers, Tamer H. Farag, Dilruba Nasrin, William C. Blackwelder, Yukun Wu, Pedro L. Alonso, Robert F. Breiman, Richard Omore, Abu S. G. Faruque, Sumon Kumar Das, Shahnawaz Ahmed, Debasish Saha, Samba O. Sow, Dipika Sur, Anita K. M. Zaidi, Fahreen Quadri, Eric D. Mintz

**Affiliations:** 1 Center for Vaccine Development, Departments of Medicine, University of Maryland School of Medicine, Baltimore, Maryland, United States of America; 2 Department of Occupational and Environmental Health, The University of Iowa, Iowa City, Iowa, United States of America; 3 Division of Foodborne, Waterborne, and Environmental Diseases, US Centers for Disease Control and Prevention, Atlanta, Georgia, United States of America; 4 Department of Pediatrics, University of Virginia School of Medicine, Charlottesville, Virginia, United States of America; 5 Gates Foundation, Seattle, Washington, United States of America; 6 Emergent BioSolutions, Gaithersburg, Maryland, United States of America; 7 Centre de Recerca en Salut Internacional de Barcelona, Hospital Clinic (CRESIB), Universitat de Barcelona, Barcelona, Spain, and Centro de Investigação em Saúde de Manhiça, Maputo, Mozambique; 8 World Health Organization, Geneva, Switzerland; 9 US Centers for Disease Control and Prevention, Nairobi, Kenya; 10 Emory Global Health Institute, Emory University, Atlanta, Georgia, United States of America; 11 Kenya Medical Research Institute/Centers for Disease Control and Prevention (KEMRI/CDC), Kisumu, Kenya; 12 International Centre for Diarrhoeal Disease Research, Mohakhali, Dhaka, Bangladesh; 13 The University of Queensland, Brisbane, Australia; 14 Medical Research Council (United Kingdom), Fajara, The Gambia; 15 Centre pour le Développement des Vaccins, Bamako, Mali; 16 National Institute of Cholera and Enteric Diseases, Kolkata, India; 17 India Public Health Association and PATH India Office, New Delhi, India; 18 Department of Paediatrics and Child Health, the Aga Khan University, Karachi, Pakistan; Gates Foundation, Seattle, Washington, United States of America; Makerere University Medical School, UGANDA

## Abstract

**Background:**

Diarrheal disease is the second leading cause of disease in children less than 5 y of age. Poor water, sanitation, and hygiene conditions are the primary routes of exposure and infection. Sanitation and hygiene interventions are estimated to generate a 36% and 48% reduction in diarrheal risk in young children, respectively. Little is known about whether the number of households sharing a sanitation facility affects a child's risk of diarrhea. The objective of this study was to describe sanitation and hygiene access across the Global Enteric Multicenter Study (GEMS) sites in Africa and South Asia and to assess sanitation and hygiene exposures, including shared sanitation access, as risk factors for moderate-to-severe diarrhea (MSD) in children less than 5 y of age.

**Methods/Findings:**

The GEMS matched case-control study was conducted between December 1, 2007, and March 3, 2011, at seven sites in Basse, The Gambia; Nyanza Province, Kenya; Bamako, Mali; Manhiça, Mozambique; Mirzapur, Bangladesh; Kolkata, India; and Karachi, Pakistan. Data was collected for 8,592 case children aged <5 y old experiencing MSD and for 12,390 asymptomatic age, gender, and neighborhood-matched controls. An MSD case was defined as a child with a diarrheal illness <7 d duration comprising ≥3 loose stools in 24 h and ≥1 of the following: sunken eyes, skin tenting, dysentery, intravenous (IV) rehydration, or hospitalization. Site-specific conditional logistic regression models were used to explore the association between sanitation and hygiene exposures and MSD. Most households at six sites (>93%) had access to a sanitation facility, while 70% of households in rural Kenya had access to a facility. Practicing open defecation was a risk factor for MSD in children <5 y old in Kenya. Sharing sanitation facilities with 1–2 or ≥3 other households was a statistically significant risk factor for MSD in Kenya, Mali, Mozambique, and Pakistan. Among those with a designated handwashing area near the home, soap or ash were more frequently observed at control households and were significantly protective against MSD in Mozambique and India.

**Conclusions:**

This study suggests that sharing a sanitation facility with just one to two other households can increase the risk of MSD in young children, compared to using a private facility. Interventions aimed at increasing access to private household sanitation facilities may reduce the burden of MSD in children. These findings support the current World Health Organization/ United Nations Children's Emergency Fund (UNICEF) system that categorizes shared sanitation as unimproved.

## Abstract 2

In a matched case-control study, Kelly Baker and colleagues explore the association between sanitation and hygiene exposures and moderate-to-severe diarrhea in young children in Africa and South Asia.

## Editors' Summary

**Background:**

Diarrhea—passing three or more loose or liquid stools per day—is a leading cause of death among children under 5 y old. Diarrhea, which can cause severe dehydration, kills about three-quarters of a million young children every year, mainly in resource-limited countries. Frequent bouts of diarrhea also cause long-term damage to the gut, malnutrition, and growth stunting. Diarrhea is a common symptom of gastrointestinal infections. The enteric pathogens (viruses, bacteria, and parasites infecting the gut) that cause diarrhea spread through contaminated food or drinking water and through poor hygiene (for example, failure to wash one’s hands after using the toilet) and inadequate sanitation (unsafe disposal of human excreta). Improvements in water, sanitation, and hygiene can reduce exposure to enteric pathogens, thereby reducing the incidence of diarrhea among young children. For example, access to an improved sanitation facility reduces the risk of diarrhea in young children by up to 36%.

**Why Was This Study Done?:**

In 2000, world leaders agreed to reduce the proportion of the global population without access to safe drinking water and basic sanitation to half of the 1990 level by 2015 as part of Millennium Development Goal (MDG) 7; the MDGs were designed to eradicate extreme poverty globally. To measure progress towards MDG7, the WHO/UNICEF Joint Monitoring Programme (JMP) currently defines an improved sanitation facility as an unshared facility that hygienically separates human excreta from human contact (for example, a flush toilet or a pit latrine). Facilities of an improved type that are shared by multiple households are classified by the JMP as unimproved because of worries that shared facilities are less hygienic and less accessible than private household facilities. However, some experts suggest that the JMP guidelines should be changed to allow facilities shared by five or fewer households to be considered as improved facilities if they meet the other criteria for separating human excreta from human contact. But does sharing a sanitation facility affect a child’s risk of diarrhea? Here, the researchers investigate this question by analyzing data collected by the Global Enteric Multicenter Study (GEMS).

**What Did the Researchers Do and Find?:**

GEMS is a case-control study, an observational study that compares the characteristics of people with a specific disease with those of people without that disease. It collected data on 8,592 children under 5 y old with moderate-to-severe diarrhea (MSD; experiencing diarrhea at least three times in 24 h with signs of moderate-to-severe dehydration or hospitalization) and 12,390 healthy children matched for age, gender, and location at seven sites in Africa and South Asia. Most of the households (>93%) at six of the sites had access to a sanitation facility, whereas only 70% of households at the Kenyan site had access to a facility. Compared to having a private household sanitation facility, sharing a facility with three or more households significantly increased the risk of young children developing diarrhea (a significantly increased risk is unlikely to have occurred by chance) at the study sites in Kenya, Mali, Mozambique, and Pakistan. At the sites in Kenya, Mali, and Pakistan, sharing a facility with one or two households also increased MSD risk. Sharing a sanitation facility did not increase MSD risk at the sites in The Gambia, Bangladesh, or India.

**What Do These Findings Mean?:**

These findings show that sharing a sanitation facility with one or two other households is associated with an increased risk of MSD in young children. Because this was an observational study, these findings only show an association between the use of shared sanitation facilities and MSD risk; they cannot prove that using shared facilities causes diarrhea. It could be, for example, that households who decide to invest in a private sanitation facility also prioritize safe hygiene practices. The reduced risk of diarrhea in these households might then be the result of everyone washing their hands after using the toilet rather than the result of having a private latrine. Nevertheless, these findings suggest that interventions aimed at increasing access to private household sanitation facilities might reduce the global MSD burden. Moreover, they suggest that shared sanitation facilities should continue to be classified as “unimproved” for the purposes of monitoring global access to sanitation.

**Additional Information:**

This list of resources contains links that can be accessed when viewing the PDF on a device or via the online version of the article at http://dx.doi.org/10.1371/journal.pmed.1002010.

- This study is further discussed in a *PLOS Medicine* Perspective by Jonny Crocker and Jamie Bartram
- A *PLOS Medicine* Collection on water and sanitation is available
- The World Health Organization (WHO) provides information on water, sanitation, and health (in several languages) and on diarrhea
- The WHO/UNICEF JMP is the official United Nations mechanism tasked with monitoring progress toward MDG7, Target C; the JMP Update Report 2015 is available online (key facts are available in several languages through the JMP website)
- The children’s charity UNICEF, which protects the rights of children and young people around the world, provides information on water, sanitation, and health and on diarrhea (in several languages)
- The Water Supply and Sanitation Collaborative Council and the non-governmental organization Practical Action provide information and approaches and technologies for improving sanitation
- The Sanitation and Hygiene Applied Research for Equity (SHARE) contributes to knowledge about effective, sustainable, and equitable sanitation and hygiene by supporting research to improve policy and practice worldwide.
- More information about GEMS is available

## Introduction

Enteric pathogens cause approximately 1.7 billion episodes of diarrhea per year in children <5 y old and account for ~10%–15% of all deaths in this age group [1–3]. Children in developing countries are exposed early and frequently to fecally contaminated food, water, human hands, soil, and fomites. Frequent exposure to enteric pathogens can result in a high incidence of acute diarrhea and asymptomatic gut infection in young children [4], which can lead to chronic, debilitating sequelae, including environmental enteropathy of the small intestine (previously called tropical enteropathy), malnutrition, and growth stunting [5–9]. Improvements in water, sanitation, and hygiene (WASH) can reduce exposure to enteric pathogens and thereby reduce pediatric diarrheal incidence [10,11]. Access to an improved sanitation facility, in particular, is estimated to reduce a child’s risk of diarrhea by 22% to 36% [12–15], with additional potential impacts on the incidence of asymptomatic infection, enteropathy, and growth stunting [4].

Millennium Development Goal (MDG) Target 7c calls on countries to halve, by 2015, the proportion of people who lack sustainable access to basic sanitation and safe water. To measure global progress, the WHO/United Nations Children's Emergency Fund (UNICEF)_ Joint Monitoring Program (JMP) has defined an improved sanitation facility as one that hygienically separates human excreta from human contact [16]. Acceptable types of facilities include a flush or pour-flush toilet/latrine to piped sewer system or septic tank, a pit latrine, a ventilated improved pit (VIP) latrine, a pit latrine with a slab, or a composting toilet. Facilities of an improved type that are shared by more than one household are considered unimproved because of concerns that they may be less hygienic and accessible than private household facilities. Currently only 68% of the world’s population, and 38% of people living in the least developed countries, meet criteria for access to an improved sanitation facility. An estimated 946 million people still practice open defecation, and 2.4 billion use unimproved facilities [16]. In 2015, an estimated 638 million people relied on shared sanitation facilities, 398 million in urban areas and 240 million in rural areas. This represents an increase of 361 million users since 1990—from 5% of the global population to 9% in 20 y—and is expected to continue growing [16].

Some have argued that post-2015 JMP guidelines should allow facilities shared by five or fewer households (or <30 persons) to be considered improved if they meet the other criteria for separating human excreta from human contact [17]. This is based on the premise that communal facilities shared by a few households are more likely to be co-owned and may be more accessible and hygienic compared with public facilities shared by strangers. However, individuals practicing open defecation or sharing sanitation facilities experience diarrheal disease, malnutrition, and diarrhea-related mortality more often than individuals using household facilities [18–22]. Sharing a facility was associated with an increase in soil-transmitted helminth infection among young men in Egypt [23], norovirus infection on a cruise ship [24], hospital admission for diarrhea among children 12 to 59 mo of age in Brazil [25], perinatal death in Nigerian [26,27] and Jamaican infants [28], cholera in Kenyan [29] and Zambian [30] refugee camps, and increased infant and maternal mortality death rates in 193 country-level surveys collected by WHO, UNICEF, and the World Bank [31]. Evidence on how number of households sharing a latrine affects these health risks, especially in young children, is limited to one analysis of Demographic Health Surveillance data from 51 countries. This study reported that the prevalence of self-reported diarrhea was slightly increased among those sharing a toilet with 1–5 households and >5 households—even one of improved design—compared to those with private access [32].

The Global Enteric Multicenter Study (GEMS) is a matched case-control study of moderate-to-severe diarrhea (MSD) in children <5 y old in four sites in sub-Saharan Africa and three sites in South Asia [33]. GEMS collected data on, among other things, wealth, household density, and WASH facilities and practices. The primary objective of this analysis is to describe sanitation and hygiene access across the study sites and to assess sanitation and hygiene exposures as risk factors for MSD in children <5 y old enrolled in GEMS, with a specific focus on shared sanitation. To our knowledge, this is the first study to use clinically and laboratory confirmed, rather than self-reported, diarrhea to analyze the risks associated with numbers of households sharing a sanitation facility.

## Methods

### Ethical Considerations

Written informed consent was obtained from caretakers of enrolled children. The scientific and ethical review committees of each participating organization, including in-country ethics approval, and the Institutional Review Board of the University of Maryland, Baltimore, approved the protocol and consent forms (S1 Table).

### Setting

The seven GEMS sites included Basse Sante Su, The Gambia; Nyanza Province, Kenya; Bamako, Mali; Manhiça, Mozambique; Mirzapur, Bangladesh; Kolkata, India; and Karachi (Bin Qasim Town), Pakistan [33]. Two sites are located in urban centers (Mali and India), and four sites are in rural settings (The Gambia, Mozambique, Kenya, and Bangladesh), whereas the study villages in Pakistan, located on the coast approximately 20 km outside Karachi, are considered periurban. Each GEMS site was linked to a defined population under a demographic surveillance system (DSS) that visited every household 2–3 times per year to record births, deaths, and migrations.

### Study Design and Health Outcome

The GEMS is a matched case-control study in which cases were children <5 y old seeking care for MSD at one of the sentinel health centers serving the DSS at each site (S2 Table). MSD was defined as passing three or more loose stools within 24 h, in conjunction with clinical signs of moderate-to-severe dehydration (sunken eyes, loss of skin turgor, or administration of IV fluids), dysentery, or admission to a health facility. Stool specimens were collected from all children at enrollment. Control children without diarrhea were randomly selected from the DSS population within 14 d of presentation of the case and matched to the case by age, sex, and neighborhood. Detailed GEMS clinical and epidemiologic methods have been published [33,34].

### Data Collection

Case and control enrollment into GEMS took place over 36 mo from December 1, 2007, to March 3, 2011. Demographic information collected about the case or control and his/her household (defined as a group of people who share a cooking fire) included maternal education and household size (including the number of children <5 y old). Building materials and household possessions were documented as potential indicators for constructing a wealth index for each site [33]. WASH data were collected at enrollment from the caretakers of case children presenting at health facilities and at home for matched control children by means of a standardized questionnaire. Approximately 60 (range: 50–90) days after enrollment, a trained field worker visited the household of each case and control to collect follow-up health information and record WASH observations. Information on water sources, facilities to dispose of human fecal waste, and handwashing and other hygiene practices was collected at enrollment, with additional information (including direct observation of hygiene practices, latrines, and toilet facilities) recorded at the 60-d follow-up home visit.

### Sociodemographic Variables

Five sociodemographic variables were considered in this analysis as potential confounders (Table 1). A wealth index quintile (WIQ) variable was generated by principal component analysis of 13 household assets. This method has been described elsewhere [33,35]. Access to an improved water source was defined as the main source of drinking water for the household at follow-up as a public or private piped water tap, tube well, borehole, protected dug well, protected spring, or rainwater that was available every day, with a round trip time of 30 min or less to fetch water [16].

**Table 1:** Description of sociodemographic, sanitation, and hygiene variables included in this analysis.

Variable Description	Format
**Sociodemographic Characteristics**	
Wealth index quintile*	Ordinal, 1–5, 1 (poorest) to 5 (wealthiest)
Caretaker completed primary school	Binary, reference category was caretaker did not complete at least primary school
Both parents live in household	Binary, reference category was household where either the mother or father or both did not reside
≥2 children <5 y old live in household	Binary, reference category was having one child under the age of 5 in the household
Assess to improved water source	Binary, reference category was not having access to improved water source
**Sanitation and Hygiene Variables**	
Facility type	Categorical variable, respondent chose from seven facility types: flush latrine, VIP latrine, VIP latrine with water seal, traditional pit latrine, pour-flush latrine, or no access (which included open defecation, hanging latrines, and bucket latrines)
Access to any facility	Binary, reference category is no access to facility
Facility sharing	Categorical variable, responses categorized based on median number of households sharing facility with GEMS household: private household access to a facility (reference), sharing facility with 1–2 other households, sharing facility with ≥3 other households, or no access
Open disposal of child’s feces	Binary, reference is disposal in a latrine
Feces visible in defecation area	Binary, reference is no feces observed in defecation area
Feces visible in house or yard	Binary, reference is no feces observed in house/yard
Handwashing station near house/yard	Binary, reference is no handwashing station in house/yard
Soap or ash at handwashing station	Among respondents that wash hands near dwelling; binary, reference category is no soap or ash at handwashing station

Abbreviations: GEMS, Global Enteric Multicenter Study; VIP, ventilated improved pit.

*Denotes five wealth index quintiles, with 1 representing the poorest households and 5 representing the wealthiest households.

### Sanitation and Hygiene Variables

Eight sanitation and hygiene variables were explored in this paper (Table 1), including three self-reported or observed sanitation variables, two directly observed fecal contamination variables, and two directly observed handwashing variables. Sanitation variables included facility type, facility access and sharing, and disposal of child’s feces as reported by respondent. Because of the skewed distribution of the number of households sharing facilities and for comparison across sites, the highest category for numbers of households sharing a sanitation facility was categorized based on the overall median of 3.

### Statistical Analysis

Data analysis was limited to subjects for whom complete data were available on sanitation access at enrollment and follow-up. Data were analyzed using SAS version 9.3 (SAS Institute, Cary, NC). Descriptive statistics for sociodemographic and exposure variables were reported as proportions, medians, and ranges. We aimed to describe site-to-site variability in effects; therefore, we present all results stratified by site. The modeling strategy involved estimating unadjusted effects of association between sanitation and hygiene exposures and MSD and assessing two-way interactions between exposures and age, followed by selecting and including consistent sociodemographic confounders across all sites for reporting adjusted estimates of sanitation and hygiene exposures. Site-specific univariable conditional logistic regression models were used to evaluate the relationship between sanitation exposure variables and MSD. Unadjusted matched odds ratios (mORs) and 95% confidence intervals (CIs) are reported. Since risk factors are likely to be different for infants, we assessed two-way interactions between risk factors and age. There were no significant interactions with age; thus, only main effects are reported, and all models still account for the age-, sex-, and geography-matched case-control design. For many of the primary sanitation variables of interest, there were low exposure frequencies, which limited the number of variables that could be included in multivariable conditional logistic models. Therefore, we ran separate multivariable conditional logistic models for each sanitation and hygiene exposure of interest. We considered the following variables as potential confounders: WIQs, caretaker education, parental residence in the household, other young children in the household, and access to an improved water source. We assessed for confounding one at a time in each of the models by identifying significant associations with MSD and effect size changes in our estimates of sanitation and hygiene variables. Based on previous research, we considered a priori that wealth was an important epidemiological factor associated with MSD and should be included in the multivariable models to produce adjusted estimates of sanitation and hygiene exposures [22]. We aimed to present consistent results across sites, so we adjusted for these same parameters in all site-specific multivariable conditional logistic regression models. Multivariable models were assessed for collinearity using condition index diagnostics.

## Results

### Sociodemographic Characteristics of GEMS Case and Control Households by Site

Between December 2007 and January 2011, 9,439 cases and 13,129 matched controls were enrolled at the seven GEMS sites [36]. Among these, follow-up observations of WASH in the home were available for 8,592 (91.2%) cases and 12,390 (94.4%) controls. The number of households lost to follow-up was highest in Pakistan (15.3%), moderate in Gambia (9.2%), Mali (10.3%), and Mozambique (9.2%), and low in Kenya (2.9%), Bangladesh (1.5%), and India (3.1%). Wealth index was significantly associated with MSD status for three of the seven sites (Mali, India, and Pakistan). Cases in India were more likely to have caretakers with higher education. In all sites, except for India, cases were less likely to have both parents living in the home (Table 2).

**Table 2:** Sociodemographic characteristics of GEMS case and control caretakers and their children in African and Asian sites, 2007–2011.

**African sites**	**The Gambia, *n* = 2,360**		**Kenya, *n* = 3,260**		**Mali, *n* = 3,677**		**Mozambique, *n* = 1,784**	
**Variable**	**Cases, *n* = 910**	**Controls, *n* = 1,456**	**Cases, *n* = 1,419**	**Controls, *n* = 1,841**	**Cases, *n* = 1,786**	**Controls, *n* = 1,891**	**Cases, *n* = 602**	**Controls, *n* = 1,182**
	***n* (%)**	***n* (%)**	***n* (%)**	***n* (%)**	***n* (%)**	***n* (%)**	***n* (%)**	***n* (%)**
Wealth index quintile*								
1 (poorest)	176 (19)	290 (20)	239 (17)	368 (20)	327 (18)	374 (20)	138 (24)	218 (19)
2	191 (21)	286 (20)	317 (22)	395 (22)	345 (19)	397 (21)	96 (16)	239 (20)
3	172 (19)	309 (21)	368 (26)	440 (24)	346 (19)	392 (21)	122 (21)	228 (19)
4	192 (21)	277 (19)	220 (16)	258 (14)	368 (21)	378 (20)	119 (20)	243 (21)
5 (wealthiest)	176 (19)	291 (20)	274 (19)	378 (21)	400 (22)	350 (19)	110 (19)	245 (21)
Caretaker completed primary school	858 (95)	1,379 (95)	647 (46)	889 (48)	1,512 (85)	1,603 (85)	459 (79)	885(75)
Both parents live in household	638 (70)	1,106 (76)	951 (67)	1,267 (69)	1,440(81)	1,648 (87)	310(53)	634 (54)
≥2 children <5 y old live in household	857 (95)	1,392 (96)	868 (61)	1,213 (66)	1,437 (81)	1,530 (81)	346 (59)	751 (64)
Access to improved water source†	649 (72)	1,125 (77)	602 (43)	906 (49)	1,467 (82)	1,644 (87)	404 (69)	709 (60)
**Asian sites**	**Bangladesh, *n* = 3,802**		**India, *n* = 3,472**		**Pakistan, *n* = 2,621**			
**Variable**	**Cases, *n* = 1,374**	**Controls, *n* = 2,428**	**Cases, *n* = 1,505**	**Controls, *n* = 1,967**	**Cases, *n* = 996**	**Controls, *n* = 1,625**		
	***n* (%)**	***n* (%)**	***n* (%)**	***n* (%)**	***n* (%)**	***n* (%)**		
Wealth index quintile*								
1 (poorest)	287 (21)	473 (20)	391 (26)	360 (19)	218 (23)	225 (15)		
2	266 (19)	492 (20)	253 (17)	306 (16)	175 (19)	303(20)		
3	276 (20)	469 (19)	301 (20)	458 (24)	208 (22)	358 (23)		
4	282 (21)	497 (20)	267 (18)	425 (22)	179 (19)	286 (19)		
5 (wealthiest)	263 (19)	497 (20)	272 (18)	390 (20)	168 (18)	372 (24)		
Caretaker completed primary school	346 (25)	615 (25)	620 (41)	679 (35)	785(83)	1, 246 (81)		
Both parents live in household	951 (69)	1,759 (73)	1,441 (97)	1,872 (97)	882 (93)	1,486 (96)		
≥2 children <5 y old live in household	404 (29)	715 (30)	595 (40)	716 (37)	727 (77)	1,120 (73)		
Access to improved water source†	720 (52)	1,422 (59)	1,374 (93)	1,762 (91)	388 (41)	833 (54)		

Abbreviations: GEMS, Global Enteric Multicenter Study;

Demographics described as number (%) per case or control group.

*Denotes five wealth index quintiles, with 1 representing the poorest households and 5 representing the wealthiest households.

^†^Access to an improved water source defined as the main source of drinking water for the household at follow-up is either a public or private piped water tap, tube well, borehole, protected dug well, protected spring, or rainwater that is available every day, with a round trip time of 30 min or less to fetch water [16].

### Access to Sanitation Facilities

The majority of households (>93%) in Gambia, Mali, Mozambique, Bangladesh, India, and Pakistan reported access to a sanitation facility (Table 3). Kenya had the highest proportion of households without access to any facility (30% case and 29% control households). Traditional pit latrines were the most frequently reported facility type for African sites, while pour-flush toilets were more common among Asian sites. While facility types varied across sites, no specific facility type was significantly associated with MSD in any of the sites. Thus, subsequent analysis focuses on access to any type of facility and is categorized based on the number of households sharing a facility with the GEMS family.

**Table 3:** Sanitation facility characteristics in the households of case and control children enrolled in GEMS by site, 2007–2011.

**African Sites**	**The Gambia, *n* = 2,366**		**Kenya, *n* = 3,260**		**Mali, *n* = 3,677**		**Mozambique, *n* = 1,784**	
**Sanitation Facility Variables**	**Cases, % *n* = 910**	**Controls, % *n* = 1,456**	**Cases, % *n* = 1,419**	**Controls, % *n* = 1,841**	**Cases, % *n* = 1,786**	**Controls, % *n* = 1,891**	**Cases, % *n* = 602**	**Controls, % *n* = 1,182**
Waste facilities used^§^:								
Flush toilet	1 (0.1)	1 (0.07)	0	1 (0.05)	24 (1.3)	23 (1.2)	6 (1.0)	8 (0.7)
VIP latrine	1 (0.1)	1 (0.07)	84 (5.9)	133 (7.2)	3 (0.2)	2 (0.1)	42 (7.0)	74 (6.3)
VIP latrine with water seal	0	0	5 (0.4)	9 (0.5)	2 (0.1)	4 (0.2)	0	2 (0.2)
Traditional pit latrine	895 (98.4)	1,440 (98.9)	909 (64.1)	1,161 (63.0)	1,743 (97.6)	1,850 (97.8)	545 (90.5)	1,075 (90.0)
Pour-flush toilet	11 (1.2)	11 (0.8)	0	0	14 (0.8)	11 (0.6)	3 (0.5)	6 (0.5)
No facility	2 (0.2)	3 (0.2)	421 (29.7)‡	536 (29.1)‡	0	1 (0.05)	5 (0.8)	16 (1.4)
Design unclear	0	0	0	0	0	0	1 (0.2)	1 (0.08)
Facility used†	908 (99.8)	1,453 (99.8)	998 (70.3)	1,304 (70.8)^a^	1,786 (100)	1,890 (99.9)	597 (99.2)	1,166 (98.6)
Private facility	852 (93.8)	1,389 (95.6)	165 (16.5)	287 (22.0)	822 (46.0)	971 (51.4)	551 (92.3)	1,103 (94.6)
Shared facility	50 (5.5)	60 (4.1)	775 (77.7)	911 (69.9)	963 (53.9)	919 (48.6)	38 (6.4)	56 (4.8)
Median (range) of households sharing	1.5 (1–9)	3 (1–12)	2 (1–24)	2 (1–24)	3 (1–22)	3 (1–20)	1.5 (1–4)	1 (1–15)
Unknown status	6 (0.7)	4 (0.3)	58 (5.8)	107 (6.0)	1 (0.06)	0	8 (1.3)	7 (0.6)
**Asian Sites**	**Bangladesh, *n* = 3,802**		**India, *n* = 3,472**		**Pakistan, *n* = 2,621**			
**Sanitation Facility Variables**	**Cases, % *n* = 1,374**	**Controls, % *n* = 2,428**	**Cases, % *n* = 1,505**	**Controls, % *n* = 1,967**	**Cases, % *n* = 996**	**Controls, % *n* = 1,625**		
Waste facilities used^§^:								
Flush toilet	4 (0.3)	14 (0.6)	21 (1.4)	18 (0.9)	84 (8.4)	118 (7.3)		
VIP latrine	154 (11.2)	223 (9.2)	0	0	1 (0.2)	0		
VIP latrine with water seal	226 (16.4)	469 (19.3)	1 (0.07)	0	0	1 (0.06)		
Traditional pit latrine	481 (35.0)	813 (33.5)	45 (3.0)	61 (3.1)	80 (8.0)	149 (9.2)		
Pour-flush toilet	405 (29.5)	721 (29.7)	1,413 (93.9)	1,853 (94.2)	809 (81.2)	1,319 (81.2)		
No facility	104 (7.6)	188 (7.7)	25 (1.7)‡	35 (1.8)‡	22 (2.2)	35 (2.2)		
Design unclear	0	0	0	0	0	3 (0.2)		
Facility used†	1,270 (92.4)	2,240 (92.3)	1,480 (98.3)	1,932 (98.2)	974 (97.8)	1,590 (97.8)		
Private facility	827 (65.1)	1,377 (61.4)	162 (10.9)	263 (13.6)	731 (75.1)	1,330 (83.6)		
Shared facility	438 (34.5)	846 (37.8)	1,306 (88.2)	1,665 (86.2)	232 (23.8)	245 (15.4)		
Median (range) of households sharing	2 (1–19)	2 (1–31)	7 (1–50)	6 (1–50)	2 (1–20)	2 (1–9)		
Unknown status	5 (0.4)	17 (0.8)	12 (0.8)	4 (0.2)	11 (1.1)	15 (0.9)		

Abbreviations: GEMS, Global Enteric Multicenter Study; VIP, ventilated improved pit.

^†^Denotes that denominator is of all households using a facility of some type.

^a^Denotes that denominator differs from facility type because there is an observation with a missing response as to whether shared or not.

^‡^Indicates inclusion of “Other” responses, such as the use of hanging latrines or burying waste, deemed as no facility.

^§^There were no significant differences by waste facility type between case and control households at *p* ≤ 0.05 using conditional logistic regression.

The proportion of households that shared a facility with other households differed between sites. Overall, the proportion of households that shared a facility among those with access to a facility was lowest in The Gambia (6% case and 4% control households) and Mozambique (6% case and 5% control households), which are both rural sites. The highest proportions of households reporting the use of shared facilities were in the urban Indian site (87% case and 85% control households) and the rural Kenyan site (55% case and 49% control households), followed by the urban Mali site (54% case and 49% control households). Among households sharing a sanitation facility, the median number of households sharing a facility with the GEMS family was three or less for all sites, except India, where the median number of additional households sharing a facility was seven (range 1–50) for case households and six (range 1–50) for control households (Table 3).

### Associations between Sanitation and Hygiene and MSD in Young Children

None of the potential confounders assessed impacted the effect size estimates for the sanitation and hygiene variables. Two sociodemographic variables, WIQ and whether both parents resided in the home, were significantly associated with MSD in at least three of the seven sites and were included in all the adjusted models. Compared to households with private sanitation, those sharing a sanitation facility with 1–2 households demonstrated increased odds of MSD in Kenya (adjusted mOR = 1.41; 95% CI: 1.11–1.79), Mali (adjusted mOR = 1.23; 95% CI: 1.02–1.48), Bangladesh (adjusted mOR = 0.83; 95% CI: 0.70–0.99), and Pakistan (adjusted mOR = 1.58; 95% CI: 1.19–2.09) sites (Table 4). In nearly all sites, with the exception of Bangladesh, effect sizes for sharing with ≥3 households were larger than estimates of sharing with only 1–2 households. Sharing with ≥3 households was statistically significant in Kenya (adjusted mOR = 1.63; 95% CI: 1.26–2.12), Mali (adjusted mOR = 1.68; 95% CI: 1.40–2.03), Mozambique (adjusted mOR = 8.83; 95% CI: 1.01–77.17) and Pakistan (adjusted mOR = 1.82; 95% CI: 1.25–2.66). Having no access to a sanitation facility was most frequently reported in rural Kenya and was a statistically significant risk factor for MSD in children <5 y old (adjusted mOR = 1.48; 95% CI: 1.15–1.90) (Table 4).

**Table 4:** Sanitation and hygiene-specific risk factors for MSD in young children in GEMS, 2007–2011.

Sanitation Exposures by Site (Total Cases *n* = 8,592; Total Controls *n* = 12,360)	Cases (*n*)	Controls (*n*)	Unadjusted mOR (95% CI)*	Adjusted mOR (95% CI)†
**African Sites**				
**The Gambia, *n* = 2,366**				
Household access to sanitation facility				
Private household facility	852	1,389	*Ref*	*Ref*
Shares facility with 1–2 households	32	29	1.59 (0.91–2.78)	1.69 (0.96–2.97)
Shares facility with ≥3 households	18	31	1.24 (0.60–2.58)	1.26 (0.60–2.66)
No facility	2	3	0.81 (0.13–5.00)	0.81 (0.13–5.14)
Disposes of child’s feces in open	12	20	0.96 (0.45–2.07)	0.85 (0.38–1.88)
Human feces visible in defecation area	69	105	1.15 (0.79–1.69)	1.14 (0.77–1.69)
Human feces visible in house or yard	69	113	1.02 (0.70–1.50)	1.05 (0.71–1.55)
Wash hands near dwelling/yard	880	1,412	1.19 (0.46–3.02)	1.26 (0.49–3.25)
Soap or ash at hand washing station	513	802	0.91 (0.67–1.23)	0.93 (0.69–1.27)
**Kenya, *n* = 3,260**				
Household access to sanitation facility				
Private household facility	165	287	*Ref*	*Ref*
Shares facility with 1–2 households	442	548	**1.37 (1.08–1.74)**	**1.41 (1.11–1.79)**
Shares facility with ≥3 households	333	363	**1.57 (1.22–2.04)**	**1.63 (1.26–2.12)**
No facility	421	536	**1.38 (1.08–1.76)**	**1.48 (1.15–1.90)**
Disposes of child’s feces in open	606	786	1.01 (0.87–1.17)	1.02 (0.87–1.20)
Human feces visible in defecation area	501	636	1.01 (0.83–1.22)	1.02 (0.84–1.24)
Human feces visible in house or yard	114	116	1.22 (0.92–1.61)	1.23 (0.93–1.63)
Wash hands near dwelling/yard	1,419	1,840	-	-
Soap or ash at handwashing station	749	963	1.02 (0.82–1.26)	0.99 (0.80–1.23)
**Mali, *n* = 3,677**				
Household access to sanitation facility				
Private household facility	822	971	*Ref*	*Ref*
Shares facility with 1–2 households	406	433	1.09 (0.91–1.29)	**1.23 (1.02–1.48)**
Shares facility with ≥3 households	557	486	**1.36 (1.16–1.61)**	**1.68 (1.40–2.03)**
No facility	0	1	-	-
Disposes of child’s feces in open	7	3	2.25 (0.70–7.31)	2.01 (0.51–7.82)
Human feces visible in defecation area	16	4	**4.00 (1.34–11.97)**	**3.77 (1.25–11.35)**
Human feces visible in house or yard	12	4	3.00 (0.97–9.30)	2.77 (0.89–8.68)
Wash hands near dwelling/yard	1,783	1,890	0.33 (0.04–3.21)	0.23 (0.02–2.20)
Soap or ash at handwashing station	1,015	1,118	0.92 (0.79–1.08)	0.91 (0.78–1.07)
**Mozambique, *n* = 1,784**				
Household access to sanitation facility				
Private household facility	551	1,103	*Ref*	*Ref*
Shares facility with 1–2 households	31	52	1.33 (0.79–2.23)	1.36 (0.77–2.23)
Shares facility with ≥3 households	7	4	8.30 (0.96–71.34)	**8.83 (1.01–77.17)**
No facility	5	16	0.55 (0.19–1.60)	0.68 (0.25–1.84)
Disposes of child’s feces in open	23	59	0.91 (0.67–1.23)	0.65 (0.32–1.30)
Human feces visible in defecation area	177	411	0.90 (0.68–1.18)	0.90 (0.69–1.19)
Human feces visible in house or yard	9	24	0.54 (0.22–1.30)	0.54 (0.22–1.31)
Wash hands near dwelling/yard	600	1,181	-	-
Soap or ash at handwashing station	502	1,035	**0.65 (0.46–0.93)**	**0.65 (0.45–0.92)**
**Asian Sites**				
**Bangladesh, *n* = 3,802**				
Household access to sanitation facility				
Private household facility	827	1,377	*Ref*	*Ref*
Shares facility with 1–2 households	334	630	0.86 (0.73–1.01)	**0.83 (0.70–0.99)**
Shares facility with ≥3 households	104	216	0.82 (0.63–1.05)	0.80 (0.61–1.04)
No facility	104	188	0.78 (0.59–1.04)	0.76 (0.57–1.02)
Disposes of child’s feces in open	988	1,662	**1.18 (1.00–1.38)**	**1.26 (1.05–1.52)**
Human feces visible in defecation area	72	135	0.92 (0.65–1.31)	0.92 (0.64–1.31)
Human feces visible in house or yard	13	19	1.08 (0.50–2.36)	1.13 (0.52–2.46)
Wash hands near dwelling/yard	1,363	2,408	1.33 (0.61–2.94)	1.36 (0.62–2.99)
Soap or ash at handwashing station	1,354	2,395	0.90 (0.37–2.19)	0.88 (0.36–2.14)
**India, *n* = 3,472**				
Household access to sanitation facility				
Private household facility	162	263	*Ref*	*Ref*
Shares facility with 1–2 households	200	279	1.08 (0.81–1.43)	1.04 (0.78–1.39)
Shares facility with ≥3 households	1,106	1,386	1.26 (1.00–1.59)	1.16 (0.91–1.48)
No facility	25	35	1.45 (0.72–2.90)	1.25 (0.62–2.53)
Disposes of child’s feces in open	1,185	1,395	1.18 (0.97–1.42)	1.11 (0.92–1.35)
Human feces visible in defecation area	142	137	**1.61 (1.19–2.18)**	**1.53 (1.12–2.08)**
Human feces visible in house or yard	109	119	**1.44 (1.03–2.01)**	**1.43 (1.02–2.00)**
Wash hands near dwelling/yard	1,505	1,966	-	-
Soap or ash at handwashing station	801	1,122	**0.68 (0.57–0.82)**	**0.73 (0.61–0.88)**
**Pakistan, *n* = 2,621**				
Household access to sanitation facility				
Private household facility	731	1,330	*Ref*	*Ref*
Shares facility with 1–2 households	147	160	**1.72 (1.30–2.25)**	**1.58 (1.19–2.09)**
Shares facility with ≥3 households	85	85	**2.10 (1.45–3.04)**	**1.82 (1.25–2.66)**
No facility	22	35	0.97 (0.51–1.86)	0.76 (0.40–1.44)
Disposes of child’s feces in open	193	284	1.09 (0.86–1.38)	0.82 (0.63–1.07)
Human feces visible in defecation area	307	472	1.11 (0.86–1.43)	0.99 (0.76–1.29)
Human feces visible in house or yard	291	439	1.23 (0.93–1.60)	1.15 (0.87–1.52)
Wash hands near dwelling/yard	960	1,572	0.98 (0.48–1.99)	1.06 (0.51–2.19)
Soap or ash at handwashing station	524	913	**0.76 (0.61–0.94)**	0.86 (0.69–1.07)

Abbreviations: GEMS, Global Enteric Multicenter Study; MSD, moderate to severe diarrhea; CI, confidence interval; mOR, matched odds ratio; Ref, reference category

*Unadjusted mOR from bivariate analysis.

^†^Adjusted mOR, whereby all odds ratios control for wealth index and both parents living in the household.

-Maximum likelihood estimates could not be computed because of sparse data.

Disposal of children’s feces in open areas around the home was not associated with increased risk of MSD among children <5 y old, except in Bangladesh (adjusted mOR = 1.26; 95% CI: 1.05–1.52) (Table 4). Having a defecation site with observed fecal material visible was statistically associated with MSD among children <5 y old in Mali (adjusted mOR = 3.77; 95% CI: 1.25–11.35) and India (adjusted mOR = 1.53; 95% CI: 1.12–2.08). Having human feces visible in the house/yard was a statistically significant risk factor in India (adjusted mOR = 1.43; 95% CI: 1.02–2.00).

Among those who reported that they washed their hands in or near the dwelling/yard, keeping soap or ash at the handwashing area demonstrated a protective effect against MSD in children <5 y old in Mozambique (adjusted mOR = 0.65; 95% CI: 0.45–0.92) and India (adjusted mOR = 0.73; 95% CI: 0.61–0.88) (Table 4).

## Discussion

In monitoring global progress in access to improved sanitation, the WHO/UNICEF JMP classifies shared sanitation facilities as “unimproved sanitation,” based upon the premise that hygiene conditions in these types of facilities may fail to hygienically separate human excreta from human contact [16,17]. This is a controversial topic, as communal facilities are the most economical and feasible solution for providing sanitation access to the 2.5 billion people without a private facility [16,37]. They are often used in densely populated urban areas, where space is at a premium. Furthermore, shared sanitation may still represent an improvement in hygiene conditions relative to open defecation [16,17]. However, for policy makers weighing options for investing in sanitation improvement, it is equally important to understand whether shared facilities effectively achieve global health targets compared to investment in private facilities.

The limited evidence on whether shared facilities protect users from exposure to human excreta is conflicting. Shared latrines in Tanzania contained lower concentrations of *Escherichia coli*, higher concentrations of flies, and equal concentrations of helminth ova compared to private latrines [38]. Other studies found that facilities shared by even a few households are dirtier than private facilities [39–41]. A recent meta-analysis found that shared sanitation was associated with an increased risk of adverse health outcomes, including diarrhea [18]. However, shared sanitation includes many different arrangements, from just a few closely related households to publicly run facilities that serve entire neighborhoods. Some of these facilities may be better maintained than others. In acknowledgement that risks may differ among shared facilities, the JMP recently deliberated whether facilities shared by ≤5 households or ≤30 users are likely to provide hygiene conditions that could be considered “improved.” Only one other study has explored this question, and they found a slightly elevated risk of self-reported diarrhea for children in households sharing a latrine with few and with many households [32]. Otherwise, little is known about how the number of households sharing a facility influences disease risks.

Epidemiological evidence from the GEMS case-control study offers new insights into the relationship between shared sanitation access, sanitation conditions, and pediatric diarrheal disease in low-income countries. We provide compelling evidence from four of seven GEMS sites that children <5 y old living in households sharing a latrine with ≥3 households, as well as those sharing with just 1–2 households, were more likely to experience MSD than children living in households with private sanitation. While shared sanitation did not reach statistical significance as a risk factor in all sites, this was likely due to our limited statistical power. Despite this limitation, these data demonstrated consistent effects of shared sanitation across most sites as a risk factor with increased odds of MSD at higher levels of sharing. This consistency was not observed for other sanitation and hygiene variables. However, our data in Bangladesh also suggest that there are some situations in which shared sanitation does not pose higher risks than private access and that other sanitation and hygiene factors are more important in diarrheal disease transmission. Global health policy must be based upon known risks to all members of a population, ensuring equity in health for all and doing no harm [42]. While shared sanitation may not be a risk factor in some communities, our study demonstrates that many children around the world do face risks from sharing sanitation facilities, reinforcing the findings of Heijnen et al. and Fuller et al. [32,43]. Without a clearer understanding of the factors that define safe sanitation, classifying shared facilities as “improved” at this point would potentially increase the number of children at risk for sanitation-related disease transmission. We conclude that compared to private sanitation facilities, sharing facilities with 1–2 other households does not prevent children from potential exposure to human excreta, and therefore, all shared facilities should continue to be classified as “unimproved” for monitoring global sanitation access.

More research is needed to understand the risks shared facilities pose and to identify cost-effective strategies to remediate those risks. The relationships between sanitation infrastructure, human behavior, and enteric disease risk in young children are complex and poorly understood. Children could be directly infected by fecal exposure at a sanitation facility—observed fecal material was significantly associated with MSD among children in the densely populated communities in Mali and India. Yet, in some studies direct exposure at a facility was uncommon since very young children did not typically use sanitation facilities [44,45]. Infection risks for children from shared latrines more likely reflect possible exposure pathways outside of the latrine that occur as a consequence of relying on shared facilities. Poor cleanliness of a facility and sharing a facility can increase user dissatisfaction and decrease latrine use [39,40,46–48]. Households relying on shared facilities may be more likely to practice open defecation or open child feces disposal [46]. Open child feces disposal was reported frequently by GEMS caretakers, especially among those who reported shared sanitation. If open defecation or open child feces disposal is amplified in communities relying on shared sanitation, then children may face high risks of exposure to diarrheal pathogens from public domain transmission pathways, such as playing in soil or surface water outside the household [49].

GEMS findings suggest access to private household latrines can provide protective benefits against MSD, even in communities like rural western Kenya, where open defecation and open child feces disposal was common. It is unlikely that private latrine access influences whether children play outside the home or not, so this protective effect may reflect protective benefits of private household latrine access on private (domestic) exposure pathways such as contaminated drinking water, food, household play areas, or hands [49,50]. Handwashing with soap or ash, especially after toileting, is a simple and important means for protecting oneself and others from exposure to fecal matter. In two GEMS sites, the presence of soap in the handwashing area was protective against MSD. Caretakers using shared facilities may be less likely to practice hygienic behaviors, like handwashing after defecation, because of challenges in access, which could increase infection risks for their children [43]. The reverse explanation for this relationship could also be true: households that prioritize safe hygiene practices are more likely to invest in private sanitation facilities than those that do not prioritize hygiene. While GEMS data provide information about the risks of shared sanitation, the evidence remains insufficient to precisely identify fecal transmission pathways associated with shared sanitation access or to distinguish between safe and unsafe shared facilities.

Our analysis has several limitations. Most GEMS households had access to a basic sanitation facility, which limited our ability to compare the risks of shared access to open defecation. While the aim of this paper was to describe sanitation risk factors associated with MSD consistently across sites, it did not address the substantial intersite differences in how facilities were shared. For example, in India most facilities were shared, and the median number of households sharing facilities was much greater than at any other site, with greater variation amongst the households included. The classification of shared facilities did not address variations in household composition and size that are potentially important factors contributing to facility conditions [37]. GEMS did not collect data on how shared sanitation facilities were managed and maintained and whether users themselves or service providers were responsible for maintaining hygienic conditions. We did not document important determinants of facility use, such as the distance between the sanitation facility and the household or handwashing area. Additionally, we did not document how facility septage was contained and managed. Septage from private and communal facilities frequently drains (or is collected by service providers and is dumped) into nearby areas lacking safe containment capabilities, thereby increasing neighborhood-level exposure risks. Each of these factors may be important sanitation-related confounders of the effect of shared sanitation on MSD.

This study also had methodological limitations. Modeling strategies were limited by relatively low frequencies of some of the exposure categories of interest. However, effect sizes remained similar across models, suggesting that our matched study design controlled for the most important sources of bias. We selected two sociodemographic indicators, a wealth quintile index and having two parents in the home. Although wealth indices are widely used in WASH research, it may not be a robust way of adjusting for sociodemographic confounding. While we did not detect any statistically significant interactions with age, we had limited power to detect them with the low exposure frequencies in these study populations. It is possible that differences by age, or age-related etiological differences, exist, but our data were underpowered to detect them. Finally, the observational study design allows us to describe associations but does not allow us to infer causality.

Data from GEMS have highlighted the association between shared and unhygienic sanitation facilities and MSD in children and suggest multiple pathways for enteric disease transmission in households with shared sanitation. Each pathway suggests potential interventions to reduce the risks associated with shared sanitation. A previous GEMS paper found shared sanitation access to be an important contributor to MSD, after adjusting for household drinking water practices, suggesting that combination approaches are necessary [51]. While our data suggest household latrines are the safest option, well-maintained, hygienic shared facilities that consistently ensure safety and privacy and provide access to soap and water for handwashing may motivate facility usage and reduce open defecation. In one urban setting in Brazil, the provision of community-level sanitation services effectively reduced intestinal infections in young children [52]. Complementary social messaging about proper disposal of children’s feces and the importance of handwashing with soap after defecation could also help mitigate risk by reducing exposures outside of the facility. Understanding how shared sanitation affects children’s public and domestic exposure risks could improve the design of interventions, as different strategies are necessary for public versus domestic transmission pathways [49].

The acceptability of shared facilities should be carefully explored in each setting to assess the likelihood of latrine uptake, with the cost–benefit carefully weighed. The benefits for provision of communal facilities may be low, especially if they fail to provide equitable access to safe sanitation across economic and social classes. The poor, women, and children are more likely to depend upon shared facilities or open defecation and are less likely to benefit from latrine promotion programs [32,47,53–55]. The documented lack of equity in sanitation access is consistent with diarrheal disease burden, also inequitably borne by the poorest classes [54]. Sanitation facilities that ensure safe, private, and affordable sanitation access for women—the primary caretakers of children—and a space with water for personal and infant bathing could improve child feces disposal. Understanding how to cost-effectively and equitably deliver safe sanitation worldwide is particularly relevant for policy makers and public health professionals tasked with decreasing diarrhea-related morbidity and mortality in children in developing countries. More research is needed to understand the sanitation-related behaviors, exposures, and conditions that influence disease transmission in developing countries [56]. Given that the risk of disease transmission does in some circumstances increase with the number of households sharing a facility, ensuring an adequate number of functioning and hygienic facilities with ample access to soap and water is particularly critical.

## Supporting Information

S1 Table List of Institutional Review Boards that provided ethical approval for GEMS. (DOCX)

S2 Table STROBE statement checklist of items that should be included in reports of observational studies. (DOC)

## Abbreviations

CI: confidence interval
DSS: demographic surveillance system
GEMS: Global Enteric Multicenter Study
IV: intravenous
JMP: Joint Monitoring Program
MDG: Millennium Development Goal
mOR: matched odds ratio
MSD: moderate-to-severe diarrhea
UNICEF: United Nations Children's Emergency Fund
VIP: ventilated improved pit
WASH: water, sanitation, and hygiene
WIQ: wealth index quintile
