# Supplementary material for: Sanitation and Hygiene-Specific Risk Factors for Moderate-to-Severe Diarrhea in Young Children in the Global Enteric Multicenter Study, 2007–2011: Case-Control Study
Source: PLoS Med. 2016 May 3;13(5):e1002010. doi: 10.1371/journal.pmed.1002010 (PMC4854459; doi:10.1371/journal.pmed.1002010)
Supplement: S1 Table — (DOCX) [file pmed.1002010.s001.docx]

| **Institutional Review Boards of Lead Institution and Participating Site Institutions** | |
| --- | --- |
|  | University of Maryland, Baltimore, MD, USA |
|  | Kenya Medical Research Institute (KEMRI), KENYA |
|  | Centro de Investigação em Saude da Manhiça (CISM), Manhiça, MOZAMBIQUE |
|  | Medical Research Council (MRC), Basse, GAMBIA |
|  | Centre pour le Développement des Vaccins du Mali (CVD-Mali), Bamako, MALI |
|  | National Institute of Cholera and Enteric Diseases (NICED), Kolkata, West Bengal, INDIA |
|  | International Center for Diarrheal Disease Research (ICDDR,B), Mirzapur, BANGLADESH |
|  | Aga Khan University, Karachi, PAKISTAN |
| **Institutional Review Boards of other collaborating institutions** | |
|  | Cooperative Studies Program Coordinating Center, Perry Point Veterans Administration Medical Center, Perry Point, MD, USA |
|  | Program in Microbiology and Mycology, University of Chile, Santiago, CHILE |
|  | University of Virginia School of Medicine, Charlottesville, VA, USA |
|  | International Vaccine Institute, Seoul, KOREA |
|  | Institute Pasteur, Paris, FRANCE |
|  | World Health Organization, Geneva, SWIZERLAND |
|  | Center for International Health, University of Bergen, and Institute of Public Health, NORWAY |
|  | University of Goteborg, Goteborg, SWEDEN |
|  | Rollins School of Public Health, Emory University, Atlanta, GA, USA |
|  | Johns Hopkins University Bloomberg School of Public Health, Baltimore, MD, USA |
|  | University of Melbourne, Victoria, AUSTRALIA |

Supplemental Table 1. List of Institutional Review Boards that provided ethical approval for the Global Enteric Multi-center Study (GEMS).
