# Supplementary material for: Sanitation and Hygiene-Specific Risk Factors for Moderate-to-Severe Diarrhea in Young Children in the Global Enteric Multicenter Study, 2007–2011: Case-Control Study
Source: PLoS Med. 2016 May 3;13(5):e1002010. doi: 10.1371/journal.pmed.1002010 (PMC4854459; doi:10.1371/journal.pmed.1002010)
Supplement: S2 Table — (DOC) [file pmed.1002010.s002.doc]

STROBE Statement—checklist of items that should be included in reports of observational studies

|  | Item No | Recommendation |
| --- | --- | --- |
| **Title and abstract** | 1, 4-5 | (*a*) Indicate the study’s design with a commonly used term in the title or the abstract - Sanitation and Hygiene-Specific Risk Factors for Moderate-to-Severe Diarrhea in Young Children in the Global Enteric Multicenter Study, 2007-2011: Case-Control Study |
|  |  | (*b*) Provide in the abstract an informative and balanced summary of what was done and what was found |
| Introduction | | |
| Background/rationale | 6-7 | Explain the scientific background and rationale for the investigation being reported |
| Objectives | 8 | State specific objectives, including any prespecified hypotheses - The primary objective of this analysis is to describe sanitation and hygiene access across the study sites and to assess sanitation and hygiene exposures as risk factors for MSD in children <5 years old enrolled in GEMS. ǂ |
| Methods | | |
| Study design | 8-9 | Present key elements of study design early in the paper – GEMS is a complex study and several papers have already been published describing most methods. We summarize and provide contextual information about water, sanitation, and hygiene data not provided elsewhere, with references to primary GEMS publications providing more detail about design. ǂ |
| Setting | 8-9 | Describe the setting, locations, and relevant dates, including periods of recruitment, exposure, follow-up, and data collection |
| Participants | 8-9, refs | (*a*) *Case-control study*—Give the eligibility criteria, and the sources and methods of case ascertainment and control selection. Give the rationale for the choice of cases and controls  “The GEMS is a matched case-control study where cases were children <5 years old seeking care for MSD at one of the sentinel health centers serving the DSS at each site. MSD was defined as passing 3 or more loose stools within 24 hours, in conjunction with clinical signs of moderate-to-severe dehydration (sunken eyes, loss of skin turgor, or administration of intravenous fluids), dysentery, or admission to a health facility...control children without diarrhea were randomly selected from the DSS population within 14 days of presentation of the case and matched to the case by age, gender, and neighborhood.” ǂ |
|  |  | (*b*) *Case-control study*—For matched studies, give matching criteria and the number of controls per case  - see above |
| Variables | 10 | Clearly define all outcomes, exposures, predictors, potential confounders, and effect modifiers. Give diagnostic criteria, if applicable |
| Data sources/ measurement | 9-10* | For each variable of interest, give sources of data and details of methods of assessment (measurement). Describe comparability of assessment methods if there is more than one group |
| Bias | 9-10 | Describe any efforts to address potential sources of bias |
| Study size | 8 | Explain how the study size was arrived at  - described in previous publications, references 32, 33 |
| Quantitative variables | 10 | Explain how quantitative variables were handled in the analyses. If applicable, describe which groupings were chosen and why  - wealth index quintiles created from quantitative index, per common methods  - quantitative variable for number of families sharing a sanitation facility grouped into 0, 1-2, and 3 or more based upon goal to systematically analyse all sites similarly and the limited number of households sharing a facility at some sites |
| Statistical methods | 9-10 | (*a*) Describe all statistical methods, including those used to control for confounding |
|  |  | (*b*) Describe any methods used to examine subgroups and interactions |
|  |  | (*c*) Explain how missing data were addressed |
|  |  | (*d*) *Cross-sectional study*—If applicable, describe analytical methods taking account of sampling strategy |
|  |  | (*e*) Describe any sensitivity analyses |

| Results | | |
| --- | --- | --- |
| Participants | 11* | (a) Report numbers of individuals at each stage of study—eg numbers potentially eligible, examined for eligibility, confirmed eligible, included in the study, completing follow-up, and analysed |
|  |  | (b) Give reasons for non-participation at each stage |
|  |  | (c) Consider use of a flow diagram |
| Descriptive data | 11-13* | (a) Give characteristics of study participants (eg demographic, clinical, social) and information on exposures and potential confounders |
|  |  | (b) Indicate number of participants with missing data for each variable of interest  - page 11, lines 6-9 |
|  |  |  |
| Outcome data | 11-13* |  |
|  |  | *Case-control study—*Report numbers in each exposure category, or summary measures of exposure  - Number of cases and controls by exposure category shown in every table |
|  |  |  |
| Main results | 14-20 | (*a*) Give unadjusted estimates and, if applicable, confounder-adjusted estimates and their precision (eg, 95% confidence interval). Make clear which confounders were adjusted for and why they were included  - “For many of the primary sanitation variables of interest there were low exposure frequencies which limited multivariable model selection strategies. For the purposes of reporting adjusted estimates and comparability across sites, all multivariable models included wealth index and whether both parents were living in the household. Wealth index and parent status in the home were significantly associated in at least two of the seven sites.” |
|  |  | (*b*) Report category boundaries when continuous variables were categorized  - page 14 and Table 2 show distributions for number of household sharing |
|  |  | (*c*) If relevant, consider translating estimates of relative risk into absolute risk for a meaningful time period |
| Other analyses | NA | Report other analyses done—eg analyses of subgroups and interactions, and sensitivity analyses |
| Discussion | | |
| Key results | 20-25 | Summarise key results with reference to study objectives |
| Limitations | 22-23 | Discuss limitations of the study, taking into account sources of potential bias or imprecision. Discuss both direction and magnitude of any potential bias |
| Interpretation | 23-25 | Give a cautious overall interpretation of results considering objectives, limitations, multiplicity of analyses, results from similar studies, and other relevant evidence |
| Generalisability | 23-25 | Discuss the generalisability (external validity) of the study results |
| Other information | | |
| Funding | 25 | Give the source of funding and the role of the funders for the present study and, if applicable, for the original study on which the present article is based |

ǂ Terms include: Global Enteric Multi-center Study (GEMS); Demographic Surveillance System (DSS); Moderate-to-severe diarrhea (MSD).

*Give information separately for cases and controls in case-control studies and, if applicable, for exposed and unexposed groups in cohort and cross-sectional studies.

**Note:** An Explanation and Elaboration article discusses each checklist item and gives methodological background and published examples of transparent reporting. The STROBE checklist is best used in conjunction with this article (freely available on the Web sites of PLoS Medicine at http://www.plosmedicine.org/, Annals of Internal Medicine at http://www.annals.org/, and Epidemiology at http://www.epidem.com/). Information on the STROBE Initiative is available at www.strobe-statement.org.
